# Energy Deposition upon Swift Heavy Ion Impact in Silicon Nanostructures and Surfaces

**DOI:** 10.3390/ma18184230

**Published:** 2025-09-09

**Authors:** Petar Žugec, Marko Karlušić

**Affiliations:** 1 Department of Physics, Faculty of Science, University of Zagreb, Bijenička Cesta 32, 10000 Zagreb, Croatia; 2 Ruđer Bošković Institute, Bijenička Cesta 54, 10000 Zagreb, Croatia

**Keywords:** swift heavy ion, ion track, Geant4, ion irradiation, radiation hardness, nanomaterials

## Abstract

Material changes made through the energy deposition upon the swift heavy ion impact can be strongly influenced by size effects in the case of nanomaterials. In particular, the amount of energy available for material modification can be significantly reduced through dissipation via electron emission. Here, this open problem is studied using the Geant4 code with the MicroElectronics package. The impact of silicon ions with various kinetic energies (in a range between 2.8 MeV and 280 MeV) is simulated in silicon nanomaterials (nanocubes, nanowires and thin films) and on the surface of silicon. The dimensions of all studied geometries were between 5 and 100 nm. The presented results indicate that the amount of dissipated energy can be significant and should be considered when modelling changes in nanomaterials induced by swift heavy ion impacts, because primary electrons can easily escape from nanomaterials.

## 1. Introduction

According to thermal spike models, the material changes caused by the passage of the swift heavy ion (SHI) are determined by the amount of deposited energy in the wake of the SHI. These ions, named “swift” due to their high kinetic energy (typically 1 MeV/nucleon), predominantly interact with the electronic subsystem of the material [1,2,3,4]. The appearance of nanoscale inclusions of modified material made by individual SHI, commonly known as ion tracks, is, therefore, closely related to the transient density of deposited energy into the electronic subsystem. In the inelastic thermal spike model [1], this quantity appears as a source term *A*(*r,t*) and is commonly used in the following form:

(1) $$A\left(r,t\right)=bS_{e}e^{{-\left(t-t_{0}\right)}^{2}/2s^{2}}F\left(r\right)$$

Equation (1) describes the spatial and temporal evolution of deposited energy density due to electronic excitation in the wake of the SHI. This disturbance of the electronic system is described by the Gaussian time distribution and a radial distribution *F*(*r*) of the electron energy deposition in the matter. Both *t*_0_ and *s* are on the femtosecond time scale, indicating that electron cascades acquire and deposit their energy in the close chronological proximity of the SHI passage [2]. The radial distribution *F*(*r*) was given first by the Katz model and was later calculated by Waligorski et al. [3]. The normalization constant *b* ensures that integration over time and space yields the total deposited energy equal to the electronic energy loss *S_e_* of the SHI, which is most often calculated using the SRIM code [4]. More recent calculations of the radial distribution *F*(*r*) indicate good agreement with earlier works [5] and also provide energy spectra of electrons emitted from material surfaces, which can be experimentally verified. In the case of silicon, the agreement with experimental results has been shown to be very good [5].

The emergence of nanomaterials in the form of quantum dots, nanowires, thin films and, more recently, 2D materials, has also opened up new questions about ion track formation in nanomaterials [6,7,8]. Since their characteristic dimensions can be comparable to the range of primary electrons (i.e., electrons produced in direct collision with the SHI), a significant portion of deposited energy can be carried away via electrons emitted into the vacuum. Recently, it has been suggested that nanomaterials can exhibit high radiation hardness due to rapid energy dissipation, and that the formation of ion tracks could be inhibited [9]. The results of calculations by several groups supported this observation, as a significant amount of deposited SHI energy was found to be dissipating away from thin films [10,11,12,13]. Also, the density of deposited energy close to the surface was found to be less than the corresponding density in the bulk region [14].

The aim of the present work is twofold. First, we aimed to investigate the dissipation pathways of energy deposited by SHI in nanostructures, such as nanoparticles, nanowires, and thin films. Energy retention and electron emission from different silicon nanostructures are studied. This represents a significant advance from our previous work, in which only energy dissipation from thin graphite films and graphene was considered [12,13]. The possible effects arising from the charge state of the SHI [15] are not studied here, since this issue was tackled in our previous work [12]. In the second part, energy retention and electron emission from the silicon surface are investigated, which occur in the case of grazing incidence SHI irradiation. Previously, only normal incidence irradiation has been considered [14], or ad hoc solutions have been implemented [16]. Since grazing incidence SHI irradiation has emerged as an attractive approach for patterning surfaces [16,17,18,19,20,21], for any modelling effort within thermal spike frameworks coupled with molecular dynamics simulations, knowledge of energy pathways is crucial.

## 2. Simulation Method

We use the Geant4 toolkit for the simulation of passage of particles through matter [22,23,24], version 11.02.0. In contrast to other, similar codes such as TREKIS [10,11], Geant4 offers ease of code modification and adaptability to specific simulation needs. For a high-precision transport of ions and electrons through silicon, we rely on the MicroElectronics models for the microdosimetry simulations in silicon [25,26,27], which are applicable to very low energies, which are not covered (adequately or at all) by the alternative Geant4 electromagnetic models. The MicroElectronics model’s tracking range spans from 50 keV/nucleon to 10 GeV/nucleon for ions and from 16.7 eV to 100 MeV for electrons.

**Figure 1:**
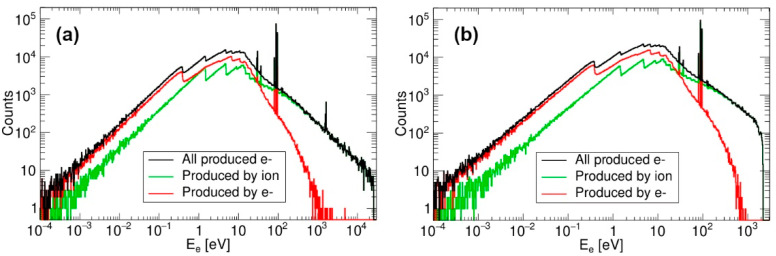
Spectra of electrons produced by 1000 impacts of (**a**) 555 MeV Ti and (**b**) 28 MeV Si in silicon.

The default Geant4 electromagnetic models implement the lower limit of 990 eV for the explicit production of electrons. Instead of generating electrons of lower energies, their energy is deposited in place. Although this limit may be manually reduced, it gives a clear sense of the limits of applicability of these default models. While the MicroElectronics energy limit of 16.7 eV (silicon plasmon energy) relates to the tracking of already produced electrons—a significant improvement over the default models—the electrons themselves may be produced even with sub-eV energies (see Figure 1). However, this mismatch between the electron production and tracking limits means that the electrons produced with energies lower than 16.7 eV are not properly propagated through the material. Instead, they are transported toward the material surface without any energy loss. In order to compensate for this clearly unrealistic feature, the immediate stopping of those electrons is implemented manually, depositing their energy in place of their production.

Since the leakage of the low-energy electrons through the material surface is of interest, a single exception to the immediate-stopping rule for <16.7 eV electrons is allowed. If the last step length towards the surface is less than 1 nm, they are allowed to reach the surface, since it is still probable that no discrete energy loss occurs along this path length, due to their extrapolated range of approximately 1 nm around 10 eV [28].

Even the MicroElectronics models do not seem to apply the appropriate work function to the electrons exiting the material. Therefore, the silicon work function of 4.8 eV [29,30] is implemented manually. If the electrons reach the surface with energy less than 4.8 eV, they are stopped and their energy is deposited within the material. In addition, when considering the total energy carried away from the material, 4.8 eV is subtracted from those electrons that do manage to leave the surface. In summary, the electrons below 4.8 eV are immediately stopped at the surface, while those above 16.7 eV always pass through. Those between 4.8 eV and 16.7 eV are allowed to exit only if their last step length is less than 1 nm.

## 3. Results and Discussion

The energy spectrum of electrons produced by the impact of 555 MeV Ti into silicon is shown in Figure 1a. Only very few electrons attain kinetic energies above 20 keV due to SHI-electron collision kinematics. Compared to the previous work where the same SHI was used [5], our result is in fair agreement. The same is shown in Figure 1b for 28 MeV Si, the SHI used in this work, in which case primary electrons (i.e., those produced in interaction with 28 MeV Si) can acquire kinetic energies up to 2 keV.

### 3.1. Silicon Nanostructures

**Figure 2:**
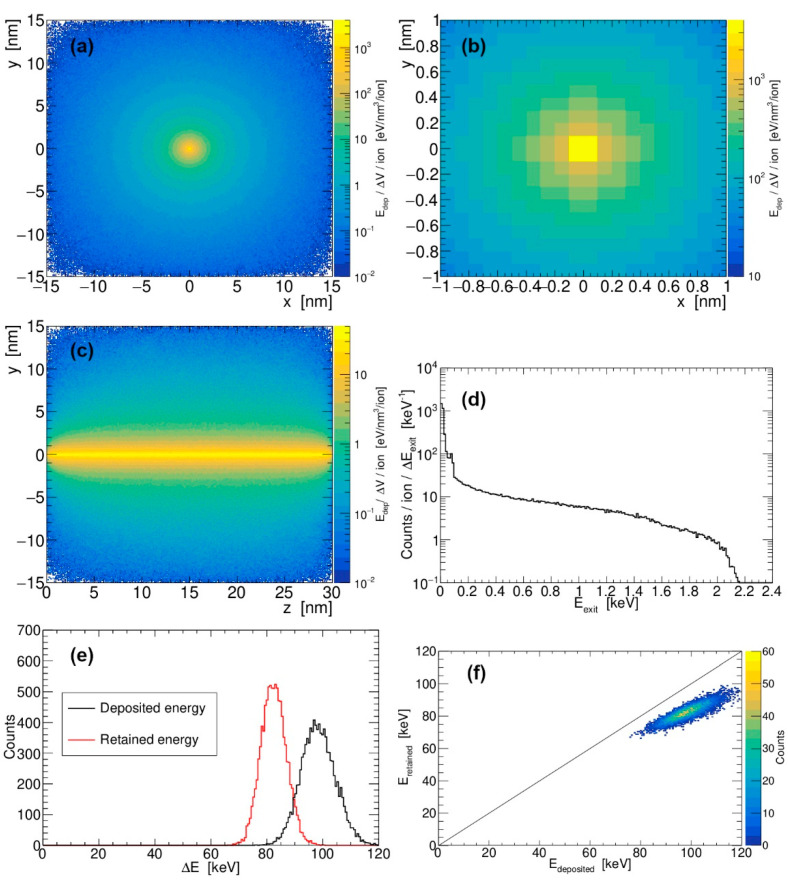
(**a**,**b**) Radial and (**c**) longitudinal profiles of the deposited energy density in the silicon nanocube (30 nm in size) after passage of 28 MeV Si. (**d**) Energy spectrum of emitted electrons generated by the passage of 28 MeV Si. (**e**,**f**) Total amount of deposited and retained energy in the silicon nanocube. Distribution is generated in a simulation run with 10,000 SHIs. Black line presents limiting case when all deposited energy is retained within the material.

In the following, the results of our calculations for energy retention and electron emission from various silicon nanostructures such as nanocube, nanowire, and thin film are presented. The results of our simulations, in which a cubic silicon nanoparticle (30 nm in size) is considered and silicon ion with a specific kinetic energy is chosen (28 MeV Si), are shown in Figure 2. Radial and longitudinal profiles of the deposited energy density are shown in Figure 2a,c, together with a zoom-in of the radial profile in Figure 2b. Energy spectra of the produced electrons shown in Figure 2d drop sharply beyond 2 keV, as expected for the collision of electrons with 1 MeV/u SHI.

Next, the pathways of excited electrons are analyzed, and the flow of energy is investigated. One part of the deposited energy is retained within the silicon nanocube, while the other part is carried away by the emitted electrons, which overcame silicon’s work function. Already in this case, for the impact of 28 MeV Si into the silicon nanocube 30 nm in size, it can be seen that a significant amount of deposited energy dissipates away. As shown in Figure 2e, the distribution of retained energy is shifted by 16 keV with respect to the distribution of deposited energy, obtained from simulation run using 10,000 SHIs. The same is shown in Figure 2f, where a 2D plot is used to represent more detailed correlations and where a black line depicts the bulk limit, when all deposited energy remains inside the material.

**Figure 3:**
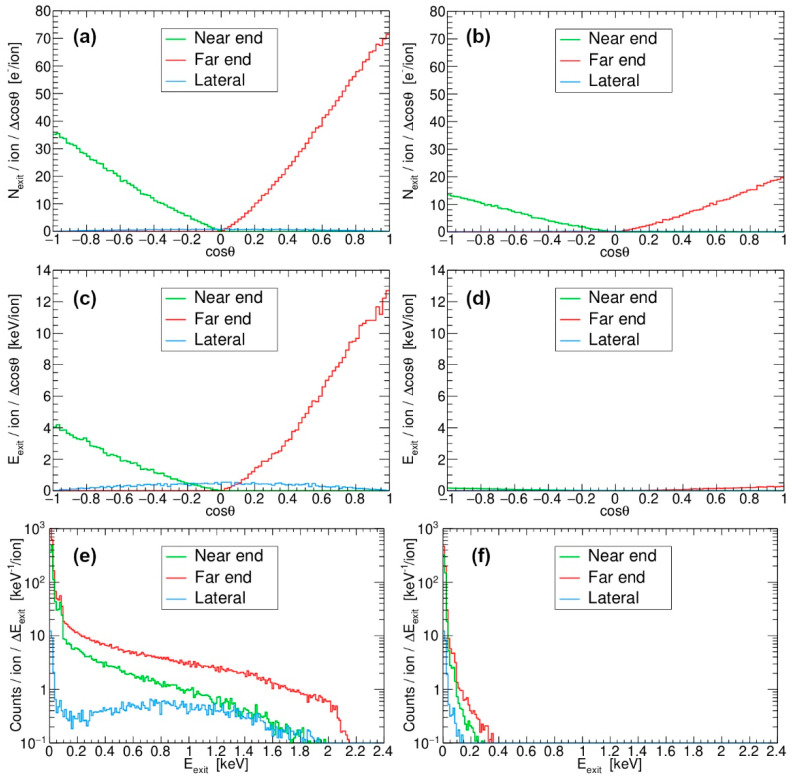
The number of (**a**) all emitted electrons and (**b**) emitted secondary electrons from the silicon nanocube 30 nm in size after 28 MeV Si impact as a function of the exit angle θ. Total energy carried away by (**c**) all emitted electrons and (**d**) emitted secondary electrons only, as a function of the exit angle θ. Energy spectra of (**e**) all emitted electrons and (**f**) emitted secondary electrons only. “Near end” is the side of the nanocube where SHI impact occurs, and “far end” is the side where SHI exits the nanocube.

The current analysis (nanocube 30 nm in size, 28 MeV Si, 10,000 SHIs) is continued by investigating electrons emitted into the vacuum. As shown in Figure 3, for each side of the nanocube, the number of emitted electrons, total emitted energy, and emitted electron energy spectra are presented. Since these quantities are experimentally accessible, data are presented in a way that is most useful, as a function of the exit angle. Graphs in Figure 3a,c,e are given for all emitted electrons and in Figure 3b,d,f for emitted secondary electrons only.

Several observations can be made about Figure 3. The number of emitted electrons shown in Figure 3a is greatest from the far side of the nanocube where the SHI exits, i.e., along the SHI movement. This is due to the SHI-electron collision kinematics, which produces primary electrons preferably in the forward direction [6]. The number of electrons emitted from the side of the cube where the SHI impacts (i.e., near end) is smaller but is still much greater than the number of electrons emitted sideways (i.e., lateral). Secondary electrons, which are present in the electron cascades, show similar behavior, as shown in Figure 3b, but their numbers are much smaller. The angular dependence of the total energy carried away shown in Figure 3c closely follows the angular dependence of the number of emitted electrons, but energy carried away by the secondary electrons is negligible (Figure 3d). This means that almost all energy that is leaving the nanocube is carried away by the primary electrons. Energy spectra of the emitted electrons shown in Figure 3e,f also confirm this observation, as a large majority of the emitted secondary electrons have less than 100 eV kinetic energy.

**Figure 4:**
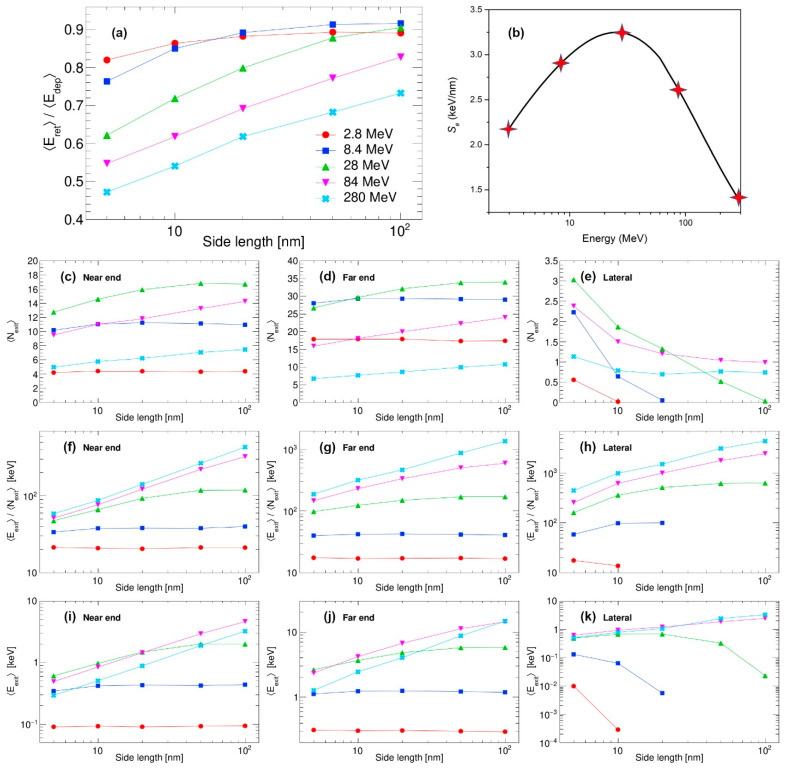
Interaction of swift silicon ions with nanocubes: (**a**) ratios of retained and deposited energy averages, (**b**) electronic energy loss of used swift silicon ions in silicon calculated by the SRIM 2013 code [4], (**c**–**e**) numbers of emitted electrons, (**f**–**h**) average energies of individual emitted electrons, and (**i**–**k**) total energies released via emitted electrons.

Finally, the raw data (such as those presented in Figure 2 and Figure 3 for the nanocube 30 nm in size and 28 MeV Si impact) are produced for all other remaining combinations of nanocube sizes (5 nm, 10 nm, 20 nm, 50 nm and 100 nm) and SHI energies (2.8 MeV, 8.4 MeV, 84 MeV and 280 MeV). These results are compiled in Figure 4, which is of our main interest. Here, in Figure 4a, we present the ratios of retained and deposited energies for different combinations of the nanocube sizes and SHI energies. It can be observed clearly that with increasing SHI energy, the percentage of retained energy diminishes, except for the lowest Si energy. For the highest SHI energy, significant dissipation of energy is found even in the largest nanocube that is 100 nm in size. Clearly, the percentage of retained energy does not depend on the electronic energy loss *S_e_* of the SHI, which is a non-linear function of the SHI kinetic energy, as calculated by the SRIM 2013 code [4] and shown in Figure 4b.

Next, the properties of the emitted electrons (i.e., the number of emitted electrons, total energy carried away, and average energies of individual electrons) are shown. At first, the number of emitted electrons increases with the kinetic energy of the SHI but then decreases for the highest energies, as shown in Figure 4c–e. Therefore, the number of emitted electrons is correlated with the electronic energy loss of the SHI, i.e., with the total amount of the energy deposited in the material by the SHI. Average values of the individual electron energies, as shown in Figure 4f–h, are correlated with the kinetic energy of the SHI. With higher energy for SHI, it generates primary electrons of higher energies, which are the ones that are able to escape even the largest nanocubes and which carry away most of the emitted energy. On the other hand, low-energy SHIs generate primary electrons of lower energies, which are sometimes unable to reach the nanocube surface, or they reach the surface with very much reduced kinetic energy. We believe this is the reason why the average energy of emitted electrons flattens out with an increase in nanocube size and/or decrease in the kinetic energy of SHI. The total emitted energy depends both on the number of emitted electrons and their individual energies. Therefore, as shown in Figure 4i–k, total emitted energy is a convolution of both the SHI energy and its electronic energy loss.

**Figure 5:**
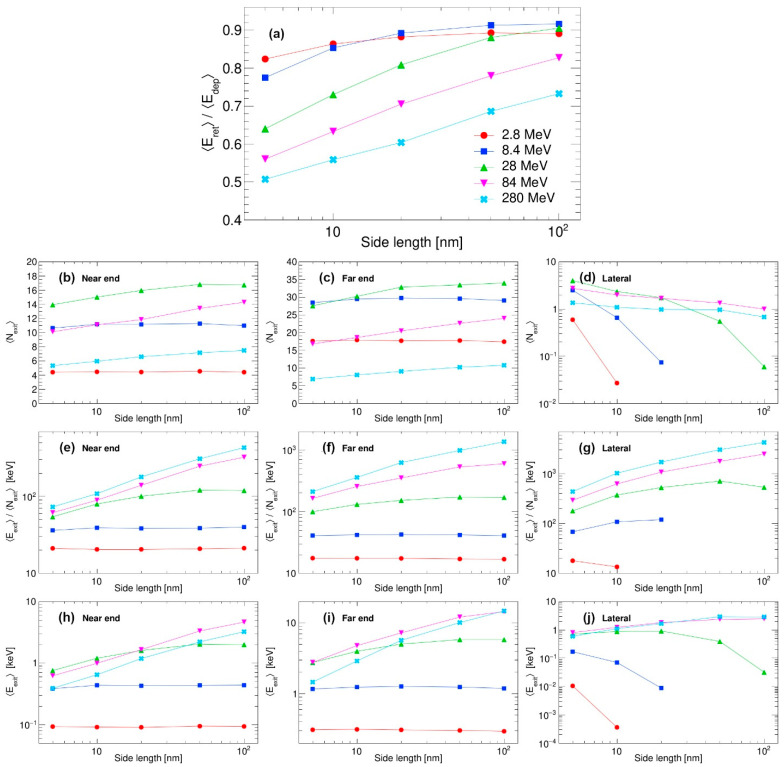
Interaction of swift silicon ions with nanowires (100 nm long with various rectangular profile sizes). (**a**) Ratios of retained and deposited energy averages, (**b**–**d**) number of emitted electrons, (**e**–**g**) average energy of individual emitted electrons, and (**h**–**j**) total energy released via the emitted electrons.

**Figure 6:**
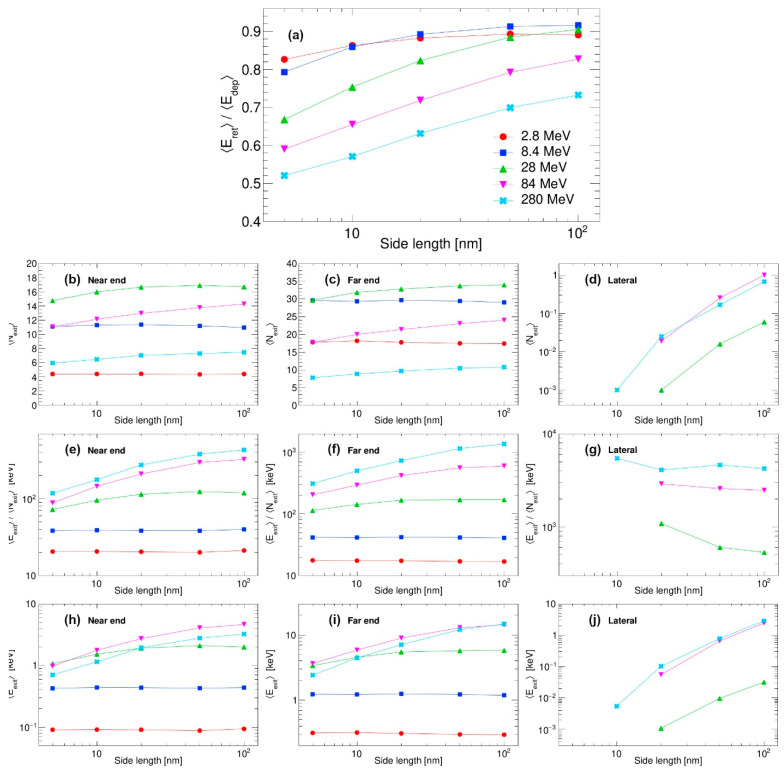
Interaction of swift silicon ions with thin films (100 nm large rectangular plate with various thicknesses). (**a**) Ratios of retained and deposited energy averages, (**b**–**d**) number of emitted electrons, (**e**–**g**) average energy of individual emitted electrons, and (**h**–**j**) total energy released via emitted electrons.

Next, we turn our attention to the interaction of SHI with other types of nanostructures. In Figure 5 and Figure 6, results similar to the results presented in Figure 4 for the nanocube are shown. In the first case, shown in Figure 5, the results of interaction of the SHI with the nanowire that is 100 nm in length and that has a rectangular profile with different sizes are shown. The SHI makes an impact in the middle of the wire’s longer side, with the SHI direction making perpendicular incidence to the wire surface. Qualitatively, the results are similar to the case of the nanocube, with just a slight increase in energy retention, as expected. The most visible changes are to the electron emission on the lateral sides, as, in this case, the length of the nanowire is fixed at 100 nm. This trend is also found in the case of thin films, when SHI makes an impact at normal incidence in the middle of the thin film, as shown in Figure 6. A slight increase in energy retention can be seen for all film thicknesses, except for the 100 nm datapoint because it corresponds to the largest nanocube and the largest nanowire. Significant changes can be seen mostly for electrons emitted from lateral sides. In that case, due to the large lateral size of the thin film, only the most energetic electrons can escape that way, and, therefore, the numbers and spectra of laterally emitted electrons are affected the most.

### 3.2. Silicon Surface

We proceed with the analysis of energy dissipation from the silicon surface when SHI irradiation is applied at the grazing incidence. In a typical experiment, SHI penetrates the surface at small angles such as 1°. In the place of the ion impact, a long ion track can be made along the irradiation direction, which can be observed easily using atomic force microscopy [16,17,18,19,31]. This type of irradiation geometry was also found to be very effective in tailoring 2D materials such as graphene [7,20]. At present, the dissipation of energy deposited close to the surface by grazing incidence SHI irradiation is an open problem [16], and energy deposition in proximity to the surface was investigated only for the normal incidence SHI irradiation [14]. This open problem is what is addressed here with a new approach using the Geant4 code.

**Figure 7:**
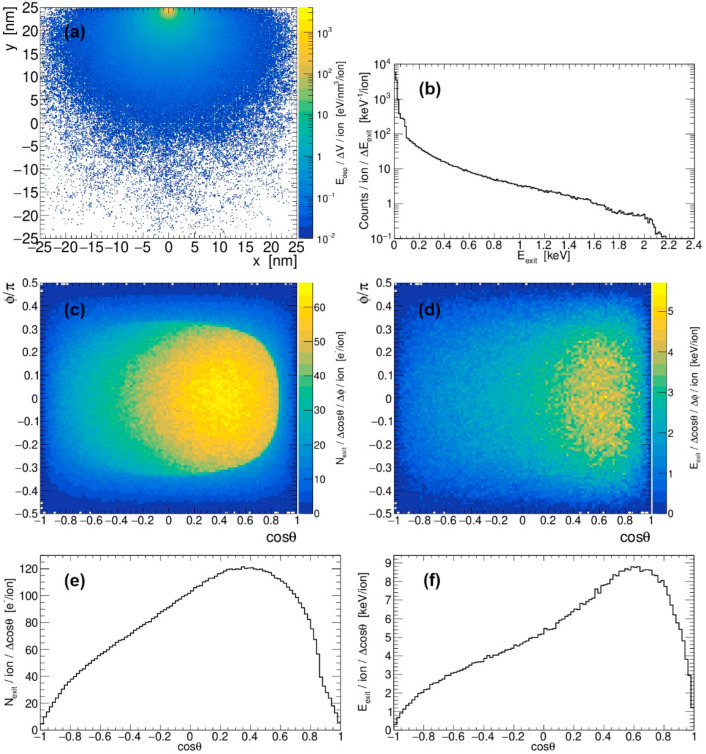
(**a**) Radial profile of the energy deposited by 28 MeV Si passing 0.5 nm below the silicon surface, constructed from the data within the 10 nm thin layer located in the middle of the target, and positioned perpendicular to the SHI trajectory. (**b**) Spectrum of electrons emitted from the silicon by 28 MeV Si impact. The 2D plot of (**c**) the number of emitted electrons and (**d**) the energy emitted from the surface after passage of 28 MeV Si ion at depth of 0.5 nm (conventional spherical system is used, with *z*-axis defined by the direction of the SHI). Angular distribution of (**e**) the number of emitted electrons and (**f**) the energy emitted from the surface under the same conditions.

Since the passage of the SHI through the material is essentially rectilinear, its movement beneath the surface can be described as a very gradual descent when irradiation is performed at grazing incidence. Thus, it is of interest to investigate the fate of deposited energy as the SHI progresses more and more in depth. This problem is tackled here by means of multiple simulation runs, choosing a nanocube 50 nm in size as a target and propagating 28 MeV Si at a certain depth, parallel to the surface. The results of our analysis, for 28 MeV Si passing 0.5 nm below the silicon surface, are presented in Figure 7.

**Figure 8:**
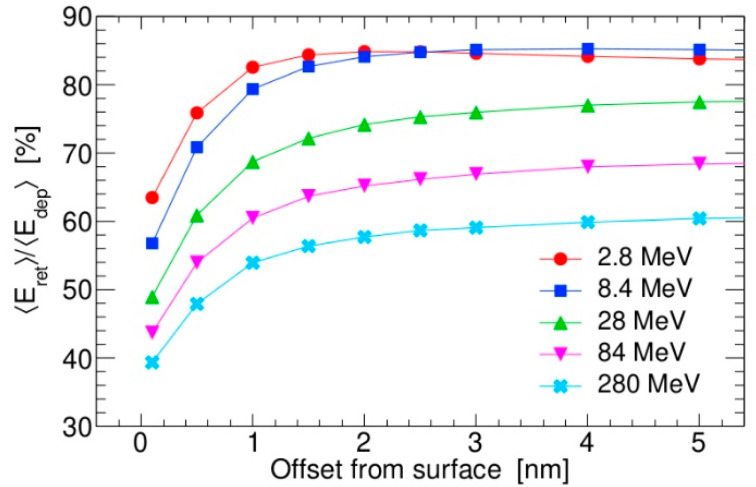
Ratios of the retained and deposited energy averages for passage of different swift silicon ions parallel to the silicon surface at various depths. Each datapoint is generated in a simulation with 1000 SHIs. Both SHI and electron deposited energy are obtained from interactions occurring within the 10 nm thin layer, located in the middle of the target and positioned perpendicular to the SHI trajectory.

Finally, in Figure 8, aggregated results are presented for the energy deposition of swift silicon ions when passing at different depths below the silicon surface. For all studied SHI energies, the influence of the surface to energy deposition is limited within the first 3 nm. This layer thickness is slightly smaller than the ~5 nm layer thickness reported by T. Ogawa et al. [14], which was obtained for SHIs with higher kinetic energies (up to 40 MeV/A) and for the normal incidence irradiation. It should be noted that retention of the energy (i.e., ratio of retained and deposited energy averages) never reaches 100% because of the finite size of the simulated target, which is a nanocube 50 nm in size. Closer to the surface, retention of the deposited energy falls even below 50% for the most energetic SHIs. Therefore, simulating ion track formation on the surface by simply removing part of the cylindrical-shaped energy density that extends above the surface might be an oversimplification [16], especially for the first ~50 nm of the ion track length made by the SHIs with kinetic energies above 1 MeV/A.

## 4. Conclusions

In this study, the results of Geant4 simulations of SHI energy deposition in silicon nanostructures (nanocube, nanowire and thin film) and on silicon surfaces are presented. These simulations cover a wide range of swift silicon ion energies (2.8 MeV–280 MeV) and silicon nanostructure sizes (5 nm–100 nm), thus offering a new and unified framework for SHI interactions with nanomaterials. Our results indicate that for the smallest nanostructures, large amounts of deposited SHI energy can be easily dissipated away via primary electron emission, regardless of the SHI energy used. The largest nanostructures show similar behavior for the highest SHI energies. This finding supports earlier observations that nanomaterials may be inherently radiation resistant because the energy deposited by the SHI impact, which is needed to modify them, cannot be effectively contained within them [9]. In addition, it is shown that energy dissipation from the surface also causes a reduced deposited energy density in the thin (~3 nm) surface layer. This feature can adversely affect the formation of surface ion tracks, especially near the SHI impact position, and should be further studied by extending the present simulations with a molecular dynamics approach.

## Data Availability

The raw data supporting the conclusions of this article will be made available by the authors on request.
